# Verbal labels increase the salience of novel objects for preschoolers with typical development and Williams syndrome, but not in autism

**DOI:** 10.1186/s11689-016-9180-7

**Published:** 2016-12-30

**Authors:** Giacomo Vivanti, Darren R. Hocking, Peter Fanning, Cheryl Dissanayake

**Affiliations:** 1 A.J. Drexel Autism Institute, Drexel University, 3020 Market Street, Suite 560, Philadelphia, PA 19104-3734 USA; 2 Olga Tennison Autism Research Centre, School of Psychology and Public Health, La Trobe University, Melbourne, Australia; 3 Developmental Neuromotor and Cognition Lab, School of Psychology and Public Health, La Trobe University, Melbourne, Australia

**Keywords:** Autism, Williams syndrome, Social learning, Referential communication

## Abstract

**Background:**

Early research has documented that young children show an increased interest toward objects that are verbally labeled by an adult, compared to objects that are presented without a label. It is unclear whether the same phenomenon occurs in neurodevelopmental disorders affecting social development, such as autism spectrum disorder (ASD) and Williams syndrome (WS).

**Methods:**

The present study used a novel eye-tracking paradigm to determine whether hearing a verbal label increases the salience of novel objects in 35 preschoolers with ASD, 18 preschoolers with WS, and 20 typically developing peers.

**Results:**

We found that typically developing children and those with WS, but not those with ASD, spent significantly more time looking at objects that are verbally labeled by an adult, compared to objects that are presented without a label.

**Conclusions:**

In children without ASD, information accompanied by the speaker’s verbal label is accorded a “special status,” and it is more likely to be attended to. In contrast, children with ASD do not appear to attribute a special salience to labeled objects compared to non-labeled objects. This result is consistent with the notion that reduced responsivity to pedagogical cues hinders social learning in young children with ASD.

## Background

Human learning is a selective process [1–3]. From infancy onward, children are more likely to pay attention to and acquire knowledge from stimuli that are accompanied by another’s communicative (“pedagogical”) cues, such as eye contact and infant-directed speech and body language [4–6]. For example, typically developing preschoolers are more likely to focus on and imitate a novel action if the model establishes mutual gaze before the demonstration [7, 8] or acts in a socially engaging manner [9, 10].

Another cue that enhances children’s attention to specific information in the environment is the presence of a verbal label. Baldwin and colleagues [11, 12] were the first to demonstrate that infants spend significantly more time looking at and manually exploring objects that are verbally labeled by an adult, compared to objects that are presented without a label. Several theoretical perspectives emphasize this as a critical process that facilitates language acquisition (see [13–15]).

The socially guided selection of relevant “to-be-learned” information reflects an early emerging sensitivity to the pedagogical structure underlying social learning. This process involves a knowledgeable adult guiding the child’s attention to relevant features in the environment through various ostensive cues, and a child equipped with dedicated cognitive resources to notice, read, and take advantage of such cues [16–18]. A corollary of this model is that children who are more socially attuned may be better equipped to access and use pedagogical information to learn from relevant aspects of the social environment.

Autism spectrum disorder (ASD) and Williams syndrome (WS) provide model disorders to investigate this notion. From infancy, children with ASD and those with WS share some overlapping deficits in the use of gesture, pointing, joint attention, and social reasoning [19–22]. However, they present with opposing profiles in their propensity to attend to and engage with their social environment, which is atypically low in ASD [23] and atypically high in WS [24]. It remains unclear how these distinct profiles of social engagement affect children’s ability to decipher and take advantage of pedagogical cues in order to select relevant “to-be-learned” information in the environment.

Children with ASD show a reduced propensity and ability to attend to and interpret social information, which impacts their ability to learn from others [25–27]. Unlike typically developing children and peers with other developmental disorders, children with ASD fail to increase their attention and spontaneous imitation in response to actions that are accompanied by eye contact [7], infant-directed body language [28], and referential looks to relevant stimuli [29–32]. Additionally, evidence suggests that children with ASD fail to use social cues such as the speaker’s referential gaze for word learning [33–35], although counter-evidence exists [36]. A recent study using eye-tracking showed that the presence of verbal labels affects attention toward novel objects in some (but not all) children with ASD [37]. Similarly, using a behavioral paradigm, McDuffie et al. [38] reported that preschoolers with ASD, just like their typically developing peers, increase their interest toward objects that are verbally labeled by an adult speaker—suggesting intact sensitivity to this pedagogical cue. However, another study [39] reported failure to increase attention to novel objects in response to verbal labels in both children with ASD and typically developing preschoolers. Conflicting results in this area of research might reflect methodological differences. In particular, the McDuffie et al. study used various manipulations, such as the use of infant-directed speech and body motion, aimed at increasing the attention of children with ASD, while other studies (e.g., [7, 32]) were designed to capture spontaneous attention and learning in situations where adults did not attempt to facilitate or enhance the child’s performance. These contrasting findings point to the need for additional research to determine the extent to which children with ASD are able to register and take advantage of different types of pedagogical cues.

Less is known about sensitivity to pedagogical cues in individuals with Williams syndrome (WS), a rare neurodevelopmental disorder (estimated prevalence of 1:7500 to 1:20,000; [40]) characterized by impaired visual-spatial abilities and social-pragmatic skills alongside an increased drive for social approach [41]. While several studies have documented increased attention and interest toward social stimuli in WS, especially toward faces and eyes [21, 42], as well as an enhanced imitation of facial expressions of emotions [43], some authors have reported diminished ability to “read” the meaning of people’s gaze and facial expressions [44, 45] and difficulties in the social-pragmatic skills needed to navigate the social environment effectively [46–48]. These findings leave open the question of how the unique social phenotype in WS affects the ability to make use of pedagogical cues to guide social learning.

The current study aimed to investigate whether verbal labels increase the salience of novel objects in young children with ASD and WS. To this end, we used a novel eye-tracking paradigm adapted from Baldwin et al.’s seminal experiment on verbal labeling [11]. We examined the extent to which preschoolers with ASD, WS, and typical development increase their visual attention to, and manual exploration of, objects that are accompanied by a verbal label relative to non-labeled objects. This paradigm afforded an exploration of the attention-facilitating effect of verbal labels in ASD, in typical development, and, for the first time, in preschoolers with WS, by taking advantage of the measurement precision provided by eye-tracking technology. Eye-tracking approaches are considered to be optimally suited for research on children with neurodevelopmental disorders who experience difficulties following verbal instructions or are distressed by social demands, such as ASD and WS [49].

Our hypothesis was that children with ASD, unlike those with WS and typically developing children, would not show more interest toward novel objects that are verbally labeled by an adult compared to objects that are presented without a verbal label. Therefore, we predicted that typically developing children and those with WS would be more inclined to pay attention to and play with objects that were labeled by an adult versus objects that were not labeled. Conversely, we predicted that propensity to pay attention to and play with objects in children with ASD would be unaffected by the presence of a verbal label. Finally, we expected a similar pattern of associations across groups, with children who are more responsive to verbal labels showing better cognitive and language skills.

## Methods

### Participants

The participants were 35 preschoolers with autism spectrum disorder (ASD), 18 preschoolers with William syndrome (WS), and 20 typically developing children (TD) who were matched on chronological age and, in the case of the ASD and WS groups, cognitive and language level. The children with ASD were recruited through the Victorian Autism Specific Early Learning and Care Centre, an intervention program located at the La Trobe University Community Children’s Centre. Participants in the WS group were recruited through the Williams Syndrome Family Support Group (Victoria) and the Williams Syndrome Association Australia, and those in the TD group were recruited through a childcare service located in the Macquarie University Campus. Parents provided informed consent after reading a participant information sheet about the study and ethics approval for the consent procedure in this study was provided by the La Trobe Human Ethics Committee (reference no. 14-007).

Diagnoses of ASD were previously made by community-based health care professionals and confirmed using the Autism Diagnostic Observation Schedule (ADOS 2, [50]) administered by a clinician with demonstrated reliability in the use of this measure. Exclusion criteria included the presence of uncorrected hearing or vision impairment and the presence of major medical problems. All participants with WS had their diagnosis confirmed with the positive fluorescent in situ hybridization (FISH) test and displayed the typical ~1.6 Mb heterozygous microdeletion at 7q11.23 [51].

Participants’ cognitive level was measured with the Mullen Scales of Early Learning (MSEL; [52]), and their adaptive behavior was assessed using the Vineland Adaptive Behavior Scales (VABS; [53]). Developmental quotient (DQ) scores were calculated according to the formula: DQ = age equivalent scores/chronological age × 100, and averaged to create an overall DQ, a verbal DQ (encompassing the receptive and expressive language subscales), and a non-verbal DQ (encompassing the visual reception and fine motor subscales). The ASD and WS groups did not differ on language or cognitive level, motor skills, and overall adaptive behavior (Table 1). However, as expected, children with WS had higher scores on the socialization subscale of the VABS compared to the children with ASD. Both clinical groups had significantly lower scores on each measure compared to children in the TD group.

**Table 1:** Participants’ characteristics

	ASD (*N* = 35)	WS (*N* = 18)	TD (*N* = 20)	*T* test *p* value ASD vs WS	*T* test *p* value ASD vs TD	*T* test *p* value WS vs TD
Age (months) *M* (SD)	45.78 (10.62)	50.00 (17.28)	50.85 (12.52)	0.35	0.10	0.50
Gender: M, F	31, 4	9, 9	13, 7	–	–	–
MSEL total DQ *M* (SD)	64.65 (30.15)	57.80 (14.17)	104.47 (13.77)	0.36	<0.001	<0.001
MSEL verbal DQ *M* (SD)	58.69 (28.53)	56.85 (15.94)	104.27 (15.98)	0.82	<0.001	<0.001
MSEL non-verbal DQ *M* (SD)	70.62 (29.23)	58.74 (13.83)	104.66 (13.87)	0.11	<0.001	<0.001
VABS communication score *M* (SD)	74.23 (20.11)	72.72 (10.54)	106.74 (16.53)	0.77	<0.001	<0.001
VABS daily leaving skill score *M* (SD)	73.29 (29.14)	71.00 (12.14)	101.05 (12.95)	0.75	<0.001	<0.001
VABS socialization score *M* (SD)	73.13 (15.15)	83.39 (12.17)	106.74 (16.53)	0.01	<0.001	<0.001
VABS motor skill score *M* (SD)	76.26 (20.35)	70.00 (10.88)	103.00 (9.46)	0.29	<0.001	<0.001
VABS ABC score *M* (SD)	71.23 (20.65)	71.39 (10.10)	103.89 (11.52)	0.97	<0.001	<0.001
ADOS social affect *M* (SD)	13.43 (4.62)	–	–	–		
ADOS repetitive behaviors *M* (SD)	4.62 (2.14)	–	–	–		

### Procedure

Participants were tested in a quiet room in one of three University or early intervention settings, depending on where the child was recruited. Three children were tested in their home. The length of experimental testing was approximately 20 min. The experiments presented here were part of a larger study.

Using a procedure inspired by Baldwin and Markman [11], we investigated whether hearing a verbal label increases the salience of novel objects through two experimental phases. First, we investigated whether participants increased their visual attention to an object that was verbally labeled by a speaker compared to objects that were accompanied by a non-verbal ostensive cue, i.e., pointing. Second, during a free play period involving the same objects that were presented previously, we measured duration of time that children manually explored the labeled and non-labeled objects.

#### Phase 1

**Fig. 1:**
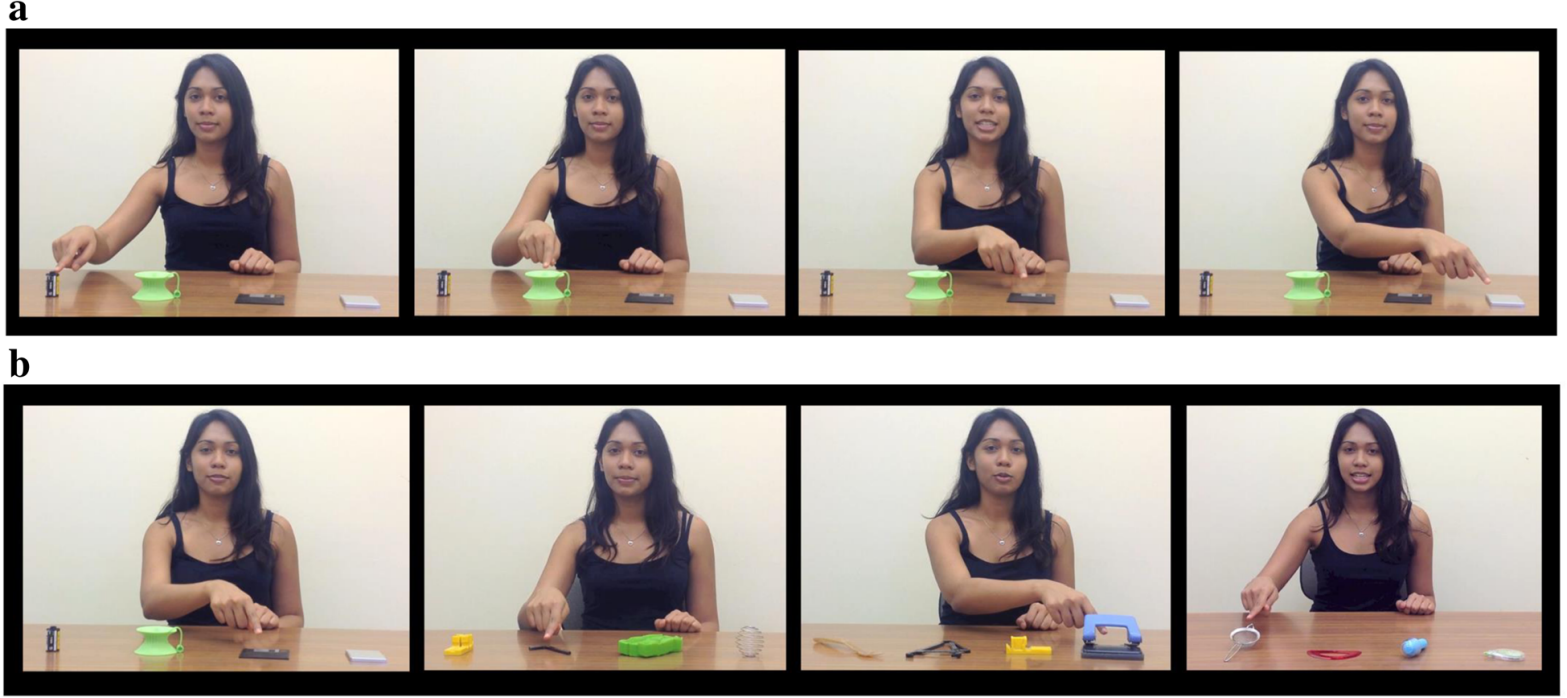
**a** Example of video-stimuli (trial 1). The actor points to each objects, adding the verbal label when pointing to the third object. **b** Sets of objects used in the four trials

Children were shown a series of four video stimuli (10 s each) on a Tobii T120 binocular eye-tracker monitor with an embedded camera (120-Hz, 1280 × 1024 pixel resolution, average precision of 0.5 of visual angle). As illustrated in Fig. 1, in each video, a female actor had a different set of four objects placed on the table in front of her. She first looked up ostensibly at the child and then pointed to each of the objects. The duration of each pointing episode was 2 s. For one object (the “labeled object”) in each video, pointing was also accompanied by a verbal label. The actor displayed neutral affect throughout the video. The 16 objects were chosen so that young children would be unfamiliar with their labels (e.g., “floppy disk,” “hole punch,” “protractor”). The position of the labeled object was different in each of the four trials. At the end of each video, the four objects were shown on a still-frame image for 4 s, which had no audio. The presentation of the video-stimuli was arranged in two fixed random orders, which were counterbalanced across participants. Videos were interspersed with filler stimuli to maintain attention.

Participants were seated in a comfortable chair, 60 cm from the computer monitor in front of a small table, with no explicit instructions given. Their attention in response to each pointing and verbal labeling episode and each pointing only episode during observation of the videos was measured to determine whether the labeled objects were attended more compared with the non-labeled objects. To this end, participants’ eye-movements were recorded using the eye-tracker system and analyzed using frame-by-frame defined areas of interest using Tobii Studio analysis software. Fixation criteria were set to Tobii Studio defaults of a 30-pixel dispersion threshold for 100 ms. The regions of interest included in the analyses were the labeled object, the non-labeled objects, and the actor’s face. Four participants in the ASD group and one in the WS group were excluded due to either a calibration error or equipment failure during the experiment.

#### Phase 2

Immediately following phase 1, participants were invited to sit on a mat and were presented with the same labeled and non-labeled objects that were previously presented in the four videos. All the objects used across the four trials were presented at the same time. The experimenter encouraged the child to play with the objects, but no specific instructions were given, and there was no active interaction with the child during this free play. In particular, the experimenter did not label any object during this phase. The duration of the free play session was 180 s. Participants’ spontaneous behavior with the objects was video-recorded, and duration of manual exploration of each object (operationalized as any episode during which children had an object in their hand for at least 1 s) was later coded by a research assistant blind to diagnostic group and study hypotheses.

## Results

### Phase 1

**Fig. 2:**
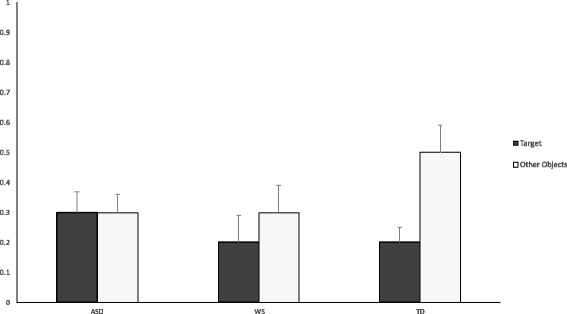
Mean fixation duration to the labeled and non-labeled objects in ASD, WS, and TD groups before the objects are labeled. *Y-axis* represents seconds

Preliminary analyses indicated that participants’ duration of attention to the objects shown in the videos was unaffected by the setting in which the experiment took place (for labeled objects *F* = 1.7, *p* = 0.2; for non-labeled objects *F* = 0.15, *p* = 0.9). Additionally, in order to rule out the possibility that the labeled-object was more visually salient than the other objects at baseline, we analyzed participants’ total duration of attention to the objects during the period of time before the actor began pointing/labeling the objects (1.5 s). This analysis showed that the labeled objects were not attended to longer than the other objects across the groups. As illustrated in Fig. 2, attention to the target object and the non-labeled objects before the actor began pointing was similar in the ASD group (*p* = 0.83) and in the WS group (*p* = 0.21), while in the TD group, there was more attention in response to non-labeled object compared to the target object (*p* < 0.05), suggesting that the labeled object was not more visually salient compared to the other objects.

**Fig. 3:**
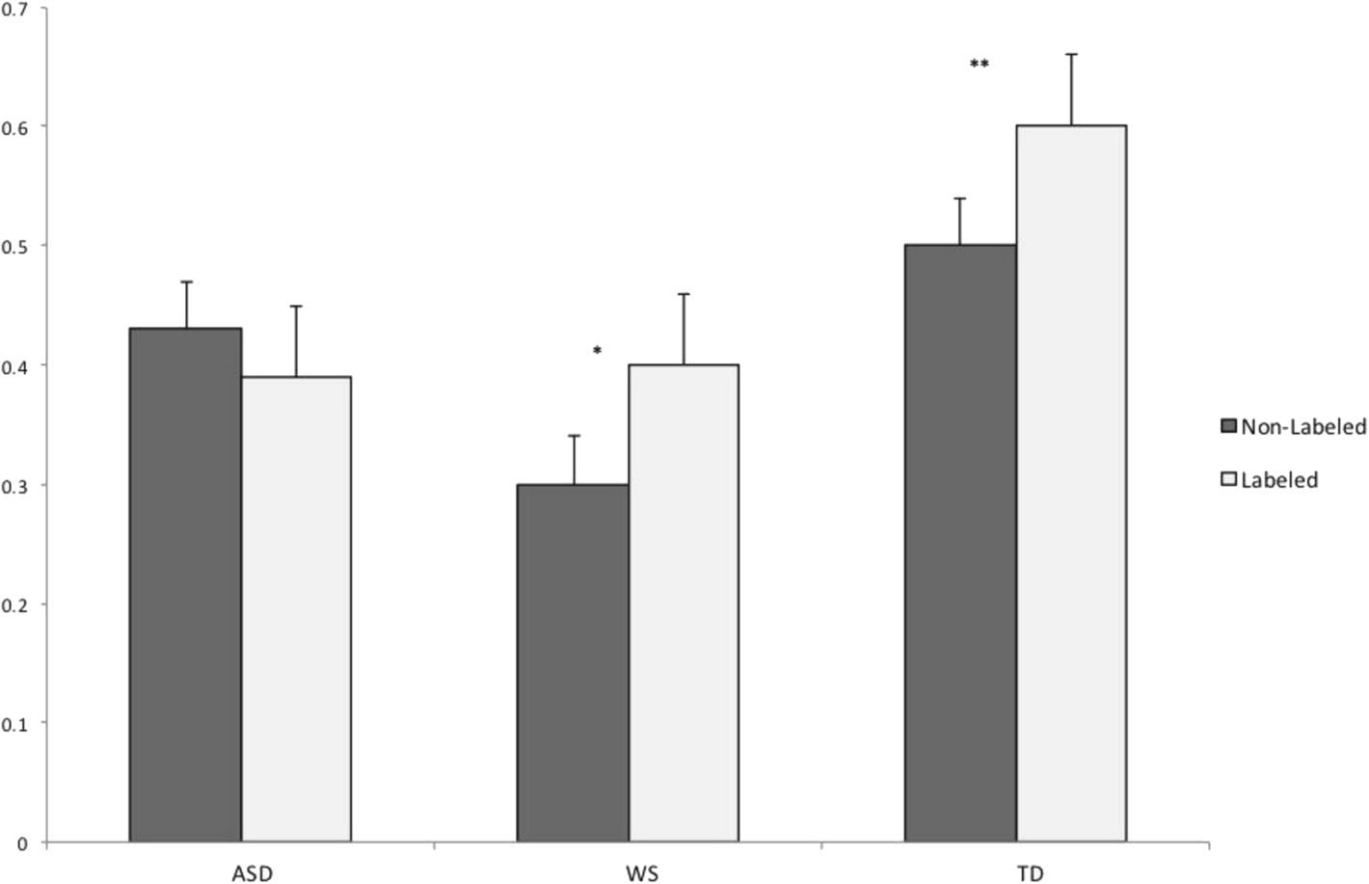
Mean fixation duration to the labeled and non-labeled objects in ASD, WS, and TD groups after the objects are labeled. *Y-axis* represents seconds. *p < 0.05, **p < 0.01

Next, participants’ visual attention to the labeled and non-labeled objects (attention duration) was subjected to a 3 (Group) × 2 (Condition-labeled, non-labeled objects) repeated measures ANOVA. Chronological age was included as a covariate term. There was no main effect of Condition (*F* (2, 64) = 1.15, *p* = 0.69, *η* ^2^_*p*_ = 0.00), a main effect of Group (*F* (2, 64) = 4.26, *p* = 0.01, *η* ^*2*^_*p*_ = 0.11), and a significant Group × Condition interaction (*F* (2, 64) = 5.28, *p* < 0.01, *η* ^*2*^_*p*_ = 0.14). There was no effect of covariate chronological age, (*F* (2, 64) = 0.07, *p* = .80, *η* ^2^_*p*_ = 0.00). As illustrated in Fig. 3, pairwise comparisons showed that while participants in the WS and TD groups looked longer to the labeled compared to the non-labeled objects (in WS adjusted *p* [Bonferroni] < 0.05, *η* ^2^_*p*_ = .06, in TD adjusted *p* [Bonferroni] < 0.01, η^2^_p_ = 0.11), this was not the case in the ASD group (adjusted *p* = 0.19, *η* ^*2*^_*p*_ = .02).

We then analyzed how participants changed their attention from the non-labeled objects prior to labeling episode to the labeled object after the object was labeled. To this aim, we used a 3 (Group) × 2 (Condition-mean fixation duration to the non-labeled objects prior to the labeling episode, mean fixation duration to the labeled object after the labeling episode) repeated measures ANOVA. Chronological age was included as a covariate term. There was an effect of Condition (*F* (2, 64) = 22.74, *p* < 0.001, *η* ^2^_*p*_ = 0.27), a main effect of Group (*F* (2, 64) = 4.29, *p* = 0.01, *η* ^2^_*p*_ = 0.12), and a significant Group × Condition interaction (*F* (2, 64) = 4.29, *p* = 0.01, *η* ^2^_*p*_ = 0.12). There was no effect of covariate chronological age, (*F* (2, 64) = 2.10, *p* = 0.15, *η* ^2^_*p*_ = 0.03). Pairwise comparisons showed that before the labeling episodes, attention to the objects was similar in the ASD group compared to the WS group (adjusted *p* [Bonferroni] = 1.00) and to the TD group (adjusted *p* [Bonferroni] = 0.18). Similarly, there was no difference between the TD and the WS group (adjusted *p* [Bonferroni] = 0.31). However, attention to the target object after the object was labeled was greater in the TD group compared to the ASD group (adjusted *p* [Bonferroni] < 0.05). Additionally, the TD group showed a trend in the same direction when compared to the WS group (adjusted *p* [Bonferroni] = 0.09). There was no significant difference between the ASD and WS group in this condition (adjusted *p* [Bonferroni] = 1.00).

Finally, participants’ visual attention to the face of the adult across conditions was subjected to a 3 (Group) × 2 (Condition-labeling, non-labeling condition) repeated measures ANOVA. Chronological age was included as a covariate term. There was no main effect of Condition (*F* (2, 64) = 0.26, *p* = 0.61, *η* ^2^_*p*_ = 0.00), Group (*F* (2, 64) = 1.88, *p* = 0.16, *η* ^2^_*p*_ = 0.05), Group × Condition interaction (*F* (2, 64) = 2.15, *p* = 0.12, *η* ^*2*^_*p*_ = .06), or covariate chronological age (*F* (2, 64) = 0.14, *p* = 0.70, *η* ^2^_*p*_ = 0.00).

### Phase 2

In order to determine whether participants explored the labeled objects more than non-labeled objects, we calculated participants’ average duration of manual exploration (number of seconds spent touching the object) of the labeled and non-labeled objects during the free play session. We then conducted a 3 (Group) × 2 (Condition-labeled objects, non-labeled objects) ANOVA. Chronological age was included as a covariate term. There was no main effect of Condition (*F* (2, 62) = 2.66, *p* = 0.11, *η* ^2^_*p*_ = 0.04), Group (*F* (2, 62) = 0.59, *p* = .55, *η* ^2^_*p*_ = 0.01), Group × Condition interaction (*F* (2, 62) = 0.59, *p* = 0.55 η^2^_*p*_ = 0.01), or covariate chronological age (*F* (2, 62) = 3.0, *p* = 0.09, *η* ^2^_*p*_ = 0.04).

Pearson correlations between visual attention and duration of manual exploration of labeled objects and measures of cognitive, social, and language abilities were examined in each group. In the ASD group, attention to the labeled objects was positively associated with verbal DQ (*r* = 0.45, *p* = 0.01) as well as Vineland Communication standard score (*r* = 0.39, *p* < 0.05) and negatively associated with severity of social symptoms (ADOS Social Affect score, *r* = −0.57, *p* = 0.001). In the WS group, there was a positive association between attention to the labeled objects and verbal DQ (*r* = 0.57, *p* = 0.01). There were no other significant associations between attention and exploration of labeled objects and any other participant characteristics.

## Discussion

In this study, we investigated whether children with ASD and WS, two neurodevelopmental disorders presenting with different patterns of atypical social attention and social learning, allocate more attentional resources to objects that are verbally labeled by a speaker compared to objects that are not labeled. To this end, we examined spontaneous visual attention and manual exploration of novel objects under conditions that varied only in the presence or absence of a verbal label. Our findings showed that hearing a label increases visual attention to unfamiliar objects in typically developing preschoolers and those with WS, but not those with ASD. This is consistent with the notion that adding a verbal label to other non-verbal indicating behaviors has an attention-facilitating effect on typical development, thus providing a pedagogical cue to guide selection of relevant information in the environment [11, 12, 54]. Additionally, these results extend our knowledge on this process in developmental disorders affecting social engagement, indicating that sensitivity and responsiveness to verbal labels is relatively preserved in WS but not in children with ASD.

Interestingly, however, these results contrast with those from a recent study by Benjamin et al. [39] who reported that typically developing preschoolers (mean age 3.5 years), as well as school-aged children with ASD (mean age 7 years), failed to increase their attention to novel objects in response to verbal labels. In the Benjamin et al. study, the labeling condition was contrasted to a condition where a speaker was talking (without labeling) about the target objects using child-directed and playful language and motion. It is possible that these cues, which were not used in the current study, caused children to pay the same amount of attention across conditions. Moreover, the children with ASD in their study were significantly older than the comparison group.

Similarly, our results contrast with those reported by McDuffie et al. [38], who documented a similar attention facilitating effect of verbal labeling in preschoolers with ASD and typical development. Importantly, methodological differences might account for the different findings. Like in the Benjamin et al. study, the non-labeling condition in the McDuffie et al. study involved a series of manipulations aimed at increasing the attention of children with ASD, including infant-directed speech (without labels) and body motion. In the current study, we opted to use a procedure inspired by the original Baldwin and Markman [11] study, which involved the use of an “emotionally neutral” adult labeling and pointing to the objects. This allowed us to evaluate whether it is verbal labeling alone which enhances children’s attention, without the potentially confounding factor of playfulness/child-directed speech.

Interestingly, however, we did not replicate one of the original findings from the Baldwin and Markman [11] study, which showed that typically developing children play for longer with labeled versus non-labeled objects. Rather, we found that participants across groups played with the objects for a similar duration of time across conditions. This finding may reflect the differences in age of participants between our studies, where Baldwin and Markman focused on infants between 10 and 20 months of age, whereas the current study examined verbal labeling in older preschoolers with typical and atypical development. Another possible explanation for this discrepancy is that one aspect of the procedure in the current study differed from the Baldwin and Markman study; in the original paradigm, labeled and non-labeled objects were presented in pairs, and the non-labeled object was not pointed to as was the case in the current study.

Importantly, our study documents for the first time that exposure to a verbal label also has a facilitative effect on attention to labeled objects in preschoolers with WS. This finding appears to be in contrast to previous reports indicating difficulties in using social-communicative cues to guide learning in children with WS. For example, a number of studies have reported difficulties in following the eye gaze of interlocutors, reduced attention to the referential targets of pointing, and reduced ability to use linguistic labels to categorize objects, as well as weaknesses in reading communicative intent in pointing, gestures, and eye gaze [55–57]. These difficulties in early social learning processes have been linked to social-communication difficulties in children with WS from infancy onward, despite their fascination for social stimuli [45, 58]. In our study, however, while communication skills (as well as cognitive abilities) in the WS group were significantly delayed and comparable to those of the ASD group, the young children with WS did not differ from age-matched typically developing children in their ability to attribute salience to verbally labeled objects. This process might be less cognitively demanding compared to the ability to read communicative facial and gestural cues. It is possible that this intact sensitivity to verbal labels facilitates language development in this population, despite the presence of cognitive and social difficulties. Further research based on longitudinal designs is needed to substantiate these speculations.

In contrast, our findings showed that preschoolers with ASD did not allocate more attentional resources to labeled versus non-labeled objects. This finding might be interpreted in the context of a diminished sensitivity to the pedagogical structure of social learning in this population [25]. Previous studies have documented that children with ASD show a diminished tendency to register, prioritize, and take advantage of the learning opportunities conveyed by cues such as pointing [59], eye contact [7], referential use of gaze [32], infant-directed speech [60], child-directed non-verbal communication [28], hearing a speaker calling one’s own name [61], and the presence of verbal labels [37]. These early emerging abnormalities have been linked to atypical language development in this population [62]. Importantly, by contrasting responses to a “pointing only” condition versus a “pointing plus labeling” condition, our findings show that deficits in ASD extend beyond difficulties in response to joint attention and involve a reduced responsivity to the “added value” of verbal labeling as an attention facilitating cue.

We found significant associations between attention to the labeled objects and measures of language and social communication in ASD. Similarly, children with WS who paid longer attention to the labeled objects had better communication skills. This finding is consistent with the notion of a link between responsiveness to the pedagogical value of verbal labels and language development and is relevant to the debate on whether language difficulties in ASD reflect specific challenges with social learning of words versus general word learning [37, 63, 64]. However, the correlational nature of the link between attentional patterns in response to labels and language proficiency documented in the current study does not provide causal information regarding the directionality of this association. Longitudinal research is now needed to study the development of the use of pedagogical cues and language development over time in typical and atypical development.

There are some limitations in the current study that should be acknowledged. One possible limitation concerns the use of video-recorded stimuli which, while allowing for rigorous eye-tracking measurement of visual attention, might be qualitatively different from the social exchanges that support processing of pedagogical cues in real life. Additionally, consistent with reports of distractibility/inattention in both children with ASD and those with WS [65, 66], the main effect of Group in the ANOVA measuring visual attention indicated that overall children in the TD groups were paying more attention to the videos compared to both the ASD and WS groups. However, there was no difference in overall attention between children with ASD and those with WS, therefore ruling out domain-general attention difficulties as an alternative explanation for why children with WS but not those with ASD increased their attention to the labeled versus non-labeled objects. An additional limitation involves the lack of inter-rater reliability procedures for the coding of manual exploration duration. Finally, the ASD group showed the gender imbalance that characterizes the ASD population, involving many more boys than girls, whereas the WS and TD groups were more gender balanced. It cannot be excluded that some of the group differences reported here are due to the fact that language development in boys progresses more slowly than in girls.

## Conclusions

In conclusion, the current study provides the first direct comparison of the facilitative effect of verbal labels in preschoolers with typical development, ASD, and WS using an eye-tracking paradigm. This methodology likely offers more precision in capturing attentional increase (or lack thereof) in response to pedagogical cues compared to behavioral coding techniques and is optimally suited for research involving children with communication difficulties such as those with ASD and WS [49]. We found that in children without ASD, information accompanied by the speaker’s verbal label is accorded a “special status,” and it is more likely to be attended to. In contrast, children with ASD do not appear to attribute a special salience to labeled objects compared to non-labeled objects.

These findings provide support to the notion that preschoolers are more likely to attend to features of the environment that are “signaled” by an adult’s pedagogical cues, a phenomenon considered to be crucial for cultural transmission, knowledge acquisition, and social development [4, 67]. Conversely, reduced responsivity to relevant communicative cues might hinder social learning in young children with ASD [25, 68]. This latter result points to the need for targeted intervention strategies that facilitate processing of pedagogical cues during early social exchanges.

## Abbreviations

ASD: Autism spectrum disorder
WS: Williams syndrome
ADOS: Autism Diagnostic Observation Schedule
MSEL: Mullen Scales of Early Learning
VABS: Vineland Scales of Adaptive Behavior
